# Supplementary material for: Effect of Fish Stock Density on Hormone Genes Expression from Brain and Gastrointestinal Tract of Salmo salar
Source: Animals (Basel). 2022 May 4;12(9):1174. doi: 10.3390/ani12091174 (PMC9102067; doi:10.3390/ani12091174)
Supplement: Supplementary file 1 [file animals-12-01174-s001.zip › animals-1666338-supplementary.pdf]

Supplementary Materials

Effect of Fish Stock Density on Hormone Genes Expression from Brain and Gastrointestinal Tract of *Salmo salar*

Claudio A. Álvarez, Paula A. Santana, Claudia B. Cárcamo, Constanza Cárdenas, Byron Morales-Lange, Felipe Ramírez, Cristian Valenzuela, Sebastián Boltaña, Javier Alcaíno, Fanny Guzmán and Luis Mercado

Supplementary Table S1. PCR primers used in this study

Gene	Genbank accession number	Primer sequence (5′-3′)	Analysis	Amplicon (bp)	E (%)	Tissue
**Neuropeptide Y (NPY)**	NM_001146681.1	**F:** TGGCCAAGTATTACTCCGCTC<br>**R:** CCTCTTCCCATACCTCTGCC	qPCR	68	95	Brain
**Calcitonin gene-related peptide (CGRP)**	NM_001146580.1	**F:** GTTTGTGCAGATGACAGCCG<br>**R:** CTGTTGCCTTCGTTGCCTTC	qPCR	63	98	Brain
**Leptin (Lp)**	FJ830677.1	**F:** GGATGATATCACGCTGCCCA<br>**R:** CACTGGACCCACACTCAGAC	qPCR	110	97	Liver
**Protachykinin-1**	MZ019485*	**F:** ATTGGTCCAAAAGAAGATCT<br>**R:** ACGAACAGAATTTTGTAACC<br>**F:** GAAATTACTTCTACCGCTTG<br>**R:** CTGGGAATTATTGGGAAAAC	Cloning	523<br>602	-	Liver
		**F:** ACAACAGAACTACAACAAGCGC<br>**R:** GACACCCCTTCCAATCTTCTGT	qPCR	107	100	
**Vasoactive intestinal peptide (VIP)**	MZ019486*	**F:** AGGGCTGGTTCGGCTGAG<br>**R:** AGAGTACCATCAGCTTAAACAGAAGAA<br>**F:**CTATATCAGTAAAAAGCTTGTTACTTCTT<br>**R:** TTGCACAAGTATACGTCATTTTATTC	Cloning	1200<br>1290	-	Intestine
		**F:** GCCACTCAGATGCCATTTTCAC<br>**R:** CTCTTTCCTGTCAGAACCGAGT	qPCR	94	99	

*Sequence characterized in this study

Supplementary Table S2. Sequences and percent identity matrix for TAC1 proteins' alignment

Number of sequence	Species	Genbank access	Aminoacid Identity (%)					
			1	2	3	4	5	6
**1**	*Salmo salar*	MZ019485*	100	89	78	99	52	48
**2**	*Danio rerio*	NP_001243320.1	78	77	100	78	48	45
**3**	*Esox lucius*	XP_010871786.1	89	100	78	89	50	49
**4**	*Salmo trutta*	XP_029588064.1	99	88	78	100	53	48
**5**	*Homo sapiens*	NP_054703.1	51	50	48	53	100	71
**6**	*Gallus gallus*	XP_004939375.2	47	48	45	48	71	100

*Sequence characterized in this study

Supplementary Table S3. Sequences and percent identity matrix for VIP precursor's alignment

Number of sequence	Species	Genbank access	Aminoacid Identity (%)					
			1	2	3	4	5	6
**1**	*Salmo salar*	MZ019486*	100	96	96	67	59	79
**2**	*Salmo trutta*	XP_029554504.1	96	100	93	66	59	78
**3**	*Esox lucius*	XP_010867977.1	96	93	100	68	61	79
**4**	*Danio rerio*	NP_001108025.2	79	78	79	63	58	100
**5**	*Homo sapiens*	NP_003372.1	67	66	68	100	55	63
**6**	*Gallus gallus*	NP_990697.2	59	59	61	55	100	58

*Sequence characterized in this study
